# Melanin-concentrating hormone promotes anxiety and intestinal dysfunction *via* basolateral amygdala in mice

**DOI:** 10.3389/fphar.2022.906057

**Published:** 2022-08-09

**Authors:** Xiaoman He, Yuhang Li, Nana Zhang, Jinfang Huang, Xing Ming, Ruixiao Guo, Yang Hu, Pengfei Ji, Feifei Guo

**Affiliations:** ^1^ Pathophysiology Department, School of Basic Medicine, Qingdao University, Qingdao, China; ^2^ Department of Clinical Laboratory, the Affiliated Hospital of Qingdao University, Qingdao, China; ^3^ Qingdao Medical College, Qingdao University, Qingdao, China

**Keywords:** basolateral amygdala, melanin-concentrating hormone, lateral hypothalamus, anxiety, intestinal dysfunction

## Abstract

The limbic system plays a pivotal role in stress-induced anxiety and intestinal disorders, but how the functional circuits between nuclei within the limbic system are engaged in the processing is still unclear. In our study, the results of fluorescence gold retrograde tracing and fluorescence immunohistochemistry showed that the melanin-concentrating hormone (MCH) neurons of the lateral hypothalamic area (LHA) projected to the basolateral amygdala (BLA). Both chemogenetic activation of MCH neurons and microinjection of MCH into the BLA induced anxiety disorder in mice, which were reversed by intra-BLA microinjection of MCH receptor 1 (MCHR1) blocker SNAP-94847. In the chronic acute combining stress (CACS) stimulated mice, SNAP94847 administrated in the BLA ameliorated anxiety-like behaviors and improved intestinal dysfunction via reducing intestinal permeability and inflammation. In conclusion, MCHergic circuit from the LHA to the BLA participates in the regulation of anxiety-like behavior in mice, and this neural pathway is related to the intestinal dysfunction in CACS mice by regulating intestinal permeability and inflammation.

## Introduction

Epidemiological studies have shown that as many as 33.7% of people are affected by anxiety disorders in their lifetimes, which causes a high disease burden (Bandelow and Michaelis, 2015). Although numerous studies have examined the brain regions involved in anxiety symptomatology, leading to the development of selective serotonin reuptake inhibitors for anxiety management, much remains unknown regarding the etiology and mechanism of anxiety (Arias et al., 2021). It is well known that long-lasting stress state caused by emotional arousal, severe trauma or intense experiences usually is a major inductive factor of psychiatric disorders, such as anxiety, depression, schizophrenia and post-traumatic stress disorder (Davis et al., 2010; Yin et al., 2016). Both animal and clinical studies found that acute or chronic stress stimuli caused morphological and functional changes in amygdala nuclei, and the hyperactivity of amygdala was considered to contribute to the anxious response (Sandi and Richter-Levin, 2009).

The amygdala, located in the medial temporal lobe of most mammals (Sah et al., 2003), plays an important role in processing information about anxiety-inducing stimuli, and modulates emotional and behavioral responses (Delgado et al., 2006; Sharp, 2017). The microcircuit in amygdala relevant for anxious responses includes the basolateral complex (BLA) and the central nucleus (CeA) (Yang and Wang, 2017; Hájos, 2021). The BLA, as the main integrated input nucleus into the amygdala, receives processed and multisensory information from different brain regions such as the sensory thalamic and cortical structures as well as the hippocampus, PFC and the locus coeruleus (McCall et al., 2015; Sah, 2017; Zhang et al., 2018; Pi et al., 2020). Neurotransmitters such as neuropeptide Y, somatostatin, cholinergic and norepinephrine also act on the BLA to modulate the behavioral manifestation of anxiety (Bedse et al., 2015; Bao et al., 2021; Gaskins et al., 2021; Sizer et al., 2021). In addition, optogenetic activation of the BLA is sufficient to induce real-time anxiety effects (Daviu et al., 2019). The CeA, mainly accepting and processing the sensory information from the BLA, is the major output region of the amygdala involved in physiological and behavioral responses to stress (Knapska et al., 2007; Namburi et al., 2015). CeA also receives minor stimuli signals from other regions(Gilpin et al., 2015), so dysfunction or activation of special receptors elicited anxiogenic-like responses (de la Mora et al., 2016; Hernandez-Perez et al., 2018; Narvaez et al., 2018; Micioni Di Bonaventura et al., 2019). Therefore, to understand anxiety disorder induced by stress, it is important to study the information input to the BLA, which might help to develop more effective therapeutics (Duval et al., 2015).

Melanin-concentrating hormone (MCH) is a brain–gut polypeptide composed of 19 amino acids. It is expressed by many cells in the lateral hypothalamic area (LHA), which is a hub integrating a variety of central and peripheral signals that coordinate adaptive behavioral responses to the environment through a complex output circuit (Chometton et al., 2016; Jimenez et al., 2018). MCHergic neurons project to entire brain regions, including the amygdala, hippocampus, and nucleus accumbens, etc., (Jang J. H. et al., 2018). In addition to being associated with feeding behavior and energy homeostasis, MCH has also been shown to play an important role in the regulation of anxiety- and depression-related behaviors in stressed rats and mice, and blocking MCH receptor 1 has antidepressant and anti-anxiety effects in various animal models (Ludwig et al., 2001; Abbott et al., 2003; Hausen et al., 2016; Jiang and Brüning, 2018). The neurochemical mechanisms underlying the regulatory role of MCH consist of decreasing the reward, inhibiting the activity of serotonergic dorsal raphe nucleus neurons, and increasing the activity of hypothalamus-pituitary adrenal axis, etc., (Shimazaki et al., 2006; Chaki et al., 2015; Torterolo et al., 2015). Injecting MCH into the cerebroventricles, locus coeruleus, or BLA has been shown to significantly decrease social interaction times, climbing times in forced swim tests, and sucrose preference in sucrose preference tests (Borowsky et al., 2002; Kim et al., 2015; Ye et al., 2018). Kim et al. (2015) 's study showed that stress can cause activation of MCH receptors in BLA, leading to defects in emotion-related behaviors. In addition, the levels of P-CamKII α in BLA of MCH KO mice were decreased, and they were able to recover from chronic stress (Kim and Han, 2016a). However, there are also studies that contradict the above results of anxiety induced by MCH, in which intranasal administration of MCH significantly reduced depression-like behaviors in stressed rats and mice (Oh et al., 2020). These findings suggest that the BLA or LHA neurons expressing MCH play a vital role in regulating anxiety and depressive behavior, while the regulation of LHA-BLA MCHergic neural pathway on anxiety remains unknown.

Stress affects gastrointestinal motility, visceral pain sensitivity, and intestinal epithelial cell permeability (O'Malley et al., 2010). Conversely, harmful colorectal dilation, which is considered a disorder of the brain–gut axis, activates the central amygdala, BLA, and anterior cingulate cortex (Tsushima et al., 2021). Although the mechanism is not clear, the close relationship between psychological stress and intestinal inflammation is widely recognized (Wang et al., 2021b). Psychological stress increases intestinal permeability and can lead to symptoms of low-grade inflammation and functional gastrointestinal diseases (Ilchmann-Diounou and Menard, 2020). Moreover, *in vitro* MCH treatment of colon cells has been shown to upregulate IL-8 transcription, suggesting a link between MCH and inflammatory pathways (Kokkotou et al., 2009).

In this study, the MCHergic neural pathway from the LHA to the BLA was activated by chemical-genetic approaches to investigate its potential effect on anxiety-like behavior and intestinal dysfunction. Chronic acute combining stress (CACS) was performed in mice to induce anxiety-like behavior, which was assessed by the open field test (OFT), elevated plus maze (EPM) test, marble burying test (MBT), and sucrose preference test (SPT). Intestinal changes were observed in CACS mice, including the motility, 5-HT and neuronal nitric oxide synthase (nNOS) expression in the myenteric plexus, intestinal inflammation, and intestinal permeability. Potential involvement of the LHA–BLA MCHergic neural pathway were investigated, and the MCH1R antagonist SNAP94847 (SNAP) was microinjected into the BLA to elucidate the involvement of LHA–BLA MCHergic signaling in brain–gut axis dysfunction in anxiety-like animal models. Present findings may contribute to the exploration of a novel treatment strategy for stress-induced anxiety and intestinal dysfunction.

## Materials and methods

### Animals

A total number of 170 male C57BL/6 mice (25–30 g) purchased from Qingdao University’s Laboratory Animal Center (Shandong, China) were used. Mice (3 per cage) were maintained under a normal 12-h light/dark cycle and had free access to tap water and standard chow. The ambient temperature and relative humidity were maintained at 24°C ± 1°C and 45% ± 5%, respectively. All animal procedures including housing, experimentation, and disposal were performed in accordance with the Guidelines for Care and Use of Laboratory Animals of Qingdao University and approved by the Animal Ethics Committee of the Medical College of Qingdao University.

### Experimental instruments and reagents

MCH (ChinaPeptides Co.,Ltd., China); Clozapine N-oxide (CNO; GLPBIO, United States); SNAP-94847 (SNAP; MedChemExpress Co., Ltd., China); AAV2/9-mMCHp-hM3D(Gq)-mCherry-WPRE-PA (Taitool Bioscience Co., Ltd., China); Stainless-steel guide cannula (model number: 62102; RWD Life Science Co. Ltd., China); Penicillin (Wuhan Biological Technology Co., Ltd., China); Fluoro-Gold (FG; Fluorochrome.LLC., United States); Microsyringe (Hamilton, Switzerland); Kryostat 1720 freezing microtome (Leica, Germany); Rabbit anti-MCHR1 (Abcam, United Kingdom); Rabbit anti-MCH (Abcam, United Kingdom); Mouse anti-c-Fos (Abcam, United Kingdom); Rat anti-5-HT (Abcam, United Kingdom); Rabbit anti-nNOS (Abcam, United Kingdom); Goat anti-mouse Cy3 (Jackson ImmunoResearch, United States); Goat anti-rabbit Alexa Fluor 488 (Jackson ImmunoResearch, United States); Antifadent mountant solutions (Citifluor, United Kingdom); DP50 digital camera (Olympus, Japan); SMART Video Tracking System (Panlab, United States); ELISA kit (Shanghai Jianglai Biotechnology Co., Ltd., China); Anti-ZO-1 (Affinity, United States); Anti-occludin (Proteintech, United States); ChemiScope 6200 system and chemical analysis software (Clinx Science Instruments, China)

### Experimental design

Experiment 1: mice were randomly divided into two groups (*n* = 10). 1.5 μl MCH (1 mg/ml) or normal saline (NS) was injected into the BLA respectively for three consecutive days through the brain cannulas. The behavioral tests were conducted 30 min after the last injection.
Experiment 2: mice were randomly selected to observe the coexistence of Fluoro-Gold and MCH immunoreactive neurons in the LHA by retrograde tracing combined with immunofluorescence histochemical staining (*n* = 6).
Experiment 3: mice were randomly divided into four groups: NS + NS, SNAP + NS, NS + CNO, and SNAP + CNO group (*n* = 6–8). The four groups were divided by intraperitoneal injection of CNO or NS and microinjection of SNAP or NS in BLA. All mice received stereotaxic injection of adeno-associated virus (AAV) vector (AAV2/9-mMCHp-hM3D(Gq)-mCherry-WPRE-PA, titer: 2E+12, 0.5 μl) in the LHA. After 2 weeks of recovery, cannula implantation were conducted in the BLA. 1 week later, 1.5 μl SNAP (2 mg/ml) or NS were microinjected in the BLA for continuous seven days. On the experiment day, CNO (0.15 mg/kg) was injected intraperitoneally to induce the activation of hM3Dq-positive MCH neurons in the LHA 20 min prior to the anxiety behaviors tests.
Experiment 4: mice were randomly subjected to the chronic unpredictable stress for 21 consecutive days and 2 h acute restraint stress on Day 22. The anxiety behaviors (*n* = 10) were monitored on Day 1, 3, 7, 14, 18, 22. Six mice were sacrificed to observe the activated MCH neurons induced by CACS on Day 22.
Experiment 5: mice were randomly divided into four groups: Control + NS group, Control + SNAP group, CACS + NS group and CACS + SNAP group. 1.5 μl SNAP (2 mg/ml) or NS was microinjected in the BLA of CACS and normal control mice respectively for consecutive seven days, and the anxiety-like behaviors (*n* = 6–10), intestinal tract motility test (*n* = 7–10), enzyme-linked immune-sorbent assay (ELISA) (*n* = 8–10), and western blot (*n* = 6) were explored respectively.

### Implantation of brain cannulas and drug microinjection

In Experiment 1, 3, 4, 5, mice were injected intraperitoneally with ketamine (100 mg/kg) and xylazine (20 mg/kg) and fixed in the stereotaxic frame. The skull was then exposed. A stainless-steel guide cannula was implanted vertically into the BLA (posterior 1.4 mm, lateral ± 3.0 mm, and depth of 4.8 mm relative to the bregma) according to the brain atlases of Paxinos and Franklin (Paxinos and Franklin, 2004) and then sealed with dental acrylic. After the operation, mice were injected with 80,000 U of penicillin (5 mg/kg) for three consecutive days to prevent infection. After 7 days recovery, mice were administered drug or vehicle in the BLA through the injection cannula connected to a syringe by a 10-cm polyethylene tube in 1.5 μl volume over 5 min. The injection cannula was kept in place for another 2 min to allow the drug to completely diffuse from the tip.

### Flouro-gold retrograde tracking

Following a previous study (Liu et al., 2020), mice were anaesthetised and fixed on the stereotaxic instrument. An aliquot of 200 nl of 2% FG was injected into BLA (posterior 1.4 mm, lateral ± 3.0 mm, and depth of 4.8 mm relative to the bregma) according to the atlas with a microsyringe. The microsyringe was kept in place for another 10 min to allow the FG to completely diffuse from the tip. Finally, the syringe was removed, and the wound was disinfected and sutured. Penicillin was injected for three consecutive days after the operation. After 7 days, the mice were anaesthetised and perfused with 4% paraformaldehyde (PFA), and their brains were removed for immunofluorescence staining.

### Immunofluorescence staining

The removed brains were postfixed for 4 h and cryoprotected in sucrose (30% in MQ water) overnight. A series of frozen coronal sections of 15 μm were cut on a freezing microtome. The slices were incubated with 4% goat serum and 0.5% Triton at room temperature for 2 h and then incubated with primary antibodies at 4°C overnight. For BLA neurons, the primary antibodies were rabbit anti-MCHR1 (1:500). For LHA neurons, the primary antibodies were rabbit anti-MCH (1:800) and mouse anti-c-Fos (1:800). For the myenteric plexus, the 1.5 cm proximal colon was taken and the mucosa, submucosa and muscularis layers of the intestine were torn out under a dissecting microscope to expose the myenteric plexus and immunofluorescence staining was performed. The primary antibodies consisted of rat anti-5-HT (1:100) and rabbit anti-nNOS (1:1,000). After washing with phosphate buffer saline (PBS) for 5 min three times, the sections were incubated with secondary antibodies, which were goat anti-mouse Cy3 (1:300), goat anti-rabbit Alexa Fluor 488 (1:100), or goat anti-rat Cy3 (1:300) at room temperature for 2 h. After rinsing with PBS three times for 5 min each wash, the sections were mounted with antifadent mountant solutions. All sections were visualized, and photographs were taken using a BX50 microscope and a DP50 digital camera. Immunoreactive cells were counted in five fields of five brain slices of the LHA of each mouse. The area of positive cell count was 350 × 350 μm^2^. The percentage of double-labeled cell was calculated as *number of double-labeled cells*/*total number of positive neurons* × 100 (Liu et al., 2020). In the myenteric plexus of each animal, 30 ganglions were randomly selected, and the immunoreactive neurons in the colon were counted. The average number of neurons in each ganglion was obtained using ImageJ software (Guo et al., 2021).

### Behavioral tests

#### Open field test

The experiment was conducted 30 min after the injection in a quiet environment with dim illumination. Each mouse was placed at the center of the bottom surface of a box and was allowed to adapt for 30 s. Its exploratory behavior was then recorded for 5 min using a camera fixed above the field. The total distance traveled and the time spent in the central area were analyzed using a SMART Video Tracking System. The inner wall and bottom of the box were cleaned with 75% ethanol after removing each animal to remove any lingering odor. The time and distance of the mice staying in the center and periphery of the open field were recorded (Matsumoto et al., 2021).

#### Elevated plus maze

A maze consisting of a plus-shaped platform with two open and two closed arms was placed 40 cm above the ground. Each mouse was placed in the maze from the center lattice to the closed arm, and its movements were recorded for 5 min. The number of times of entering the open arm and the closed arm and the residence time were taken as parameters (Wang et al., 2021a).

#### Marble burying test

The animals were transferred to a cage (47 × 25 × 30 cm) with 20 glass balls 1.5 cm in diameter arranged in a 4 × 5 grid on a 5-cm thick pad. After 30 min, the animals were placed back in their cages, and the buried marbles (up to 2/3 of their diameter) were counted (Yu et al., 2019).

#### Sucrose preference test

On the first day, the mice were acclimatized with two bottles of 1% sucrose solution for 24 h. On the second day, they were given a bottle of 1% sucrose solution and the same volume of water for 24 h. The positions of the two bottles were switched every 2 h to prevent location preferences. Sucrose preference was determined by measuring sucrose solution consumption and expressed as a percentage of the total liquids over a 4-h period (Li et al., 2021).

### Chemogenetic stimulation

In Experiment 3, the skulls of mice were exposed on the stereotaxic device after anesthesia. The bilateral stereotaxic injection of AAV2/9-mMCHp-hM3D(Gq)-mCherry-WPRE-PA was positioned at the LHA (posterior 1.34 mm, lateral ± 1.2 mm, and depth of 5.0 mm relative to the bregma) according to the atlas. After three weeks of recovery, Clozapine N-oxide (CNO) dissolved in saline (1 mg/ml) was injected (0.15 mg/kg) intraperitoneally to induce activation of hM3Dq-positive MCH neurons. Wang et al. (2021c).

### Chronic acute combining stress

In Experiment 4 and 5, mice were randomly subjected to chronic unpredictable stress for 21 consecutive days. The chronic unpredictable stress included food deprivation for 24 h, water deprivation for 24 h, swimming in cold water (4°C) for 10 min, tail pinching for 3 min, overnight illumination, and sleeping in wet cages for 6 h (Yu et al., 2019). On Day 22, mice were subjected to acute restraint stress for 2 h (Table 1).

**TABLE 1:** The protocol of chronic acute combining stress.

Days	Stressor	Days	Stressor	Days	Stressor
Day 1	sleeping in wet cages for 6 h	Day 9	swimming in cold water (4°C) for 10 min	Day 17	overnight illumination
Day 2	overnight illumination	Day 10	water deprivation for 24 h	Day 18	tail pinching for 3 min
Day 3	food deprivation for 24 h	Day 11	sleeping in wet cages for 6 h	Day 19	sleeping in wet cages for 6 h
Day 4	water deprivation for 24 h	Day 12	tail pinching for 3 min	Day 20	water deprivation for 24 h
Day 5	swimming in cold water (4°C) for 10 min	Day 13	overnight illumination	Day 21	overnight illumination
Day 6	overnight illumination	Day 14	sleeping in wet cages for 6 h	Day 22	acute restraint stress for 2 h
Day 7	tail pinching for 3 min	Day 15	swimming in cold water (4°C) for 10 min		
Day 8	food deprivation for 24 h	Day 16	food deprivation for 24 h		

### Intestinal tract motility test

#### Defecation time

The mice were fasted for 18 h, SNAP or NS were microinjected into the BLA of CACS mice and normal control mice respectively. One hour later, 0.3 ml charcoal powder (5% activated carbon dissolved in 10% acacia solution) was gavaged to the mice. The time of the first fecal ball with carbon powder was recorded.

#### Fecal moisture content test

Feces from each mouse were weighed and then dried in an oven (60°C) for 1 h. The dried feces were then weighed. The water content was calculated according to the following formula: [(*initial weight*–*dry weight*)/*initial weight*] × 100 (Yu et al., 2019; Li et al., 2021).

### Enzyme-linked immune-sorbent assay

Mice were anesthetized and proximal colon samples (0.04 g) were taken. PBS was added (360 μl), and the samples were ground on ice. After centrifugation at 1,450 × g for 20 min at 4°C, the supernatant was collected and stored at −80°C. The levels of 5-HT, nNOS, TNF-α, IL-6, and IL-10 in the samples were measured according to the ELISA kit instructions.

### Western blot

Following the method of a previous study (Cui et al., 2021), proximal colon tissue samples were collected and cleaved in RIPA lysis buffer containing phosphatase and protease inhibitors and homogenized on ice. A BCA protein detection kit was used to quantify the protein concentrations in the samples. Equivalent amounts of protein (20 μg) were analyzed on SDS-PAGE gel and then electrically transferred to a 0.45-μm PVDF membrane at room temperature and blocked in TBST with 5% skimmed milk for 2 h. It was then incubated overnight in a 4°C refrigerator with the following specific colostrum: anti-ZO-1 (1:1,000) and anti-occludin (1:3,000). The membrane was then incubated with HRP-conjugated secondary antibody (anti-rabbit/mouse IgG) for fluorescent protein band detection using a synergistic chemiluminescence solution and analyzed using a ChemiScope 6200 system and chemical analysis software.

### Histological verification

At the end of the experiment, each mouse was perfused and fixed by 4% PFA, and 50 μm frozen sections of the brains were prepared to verify the locations of nuclear injection. Incorrectly positioned data were excluded from statistical analysis.

### Statistical analysis

The data were expressed as means ± standard deviations and processed using Prism 8.0 software. Statistical significance was assessed using two-way or one-way factorial ANOVA with Bonferroni/Dunn correction for multiple comparisons and a paired *t*-test. Values of *p* < 0.05 were considered statistically significant.

## Results

### Melanin-concentrating hormone microinjection into the basolateral amygdala induced anxiety-like behaviors in mice

**FIGURE 1:**
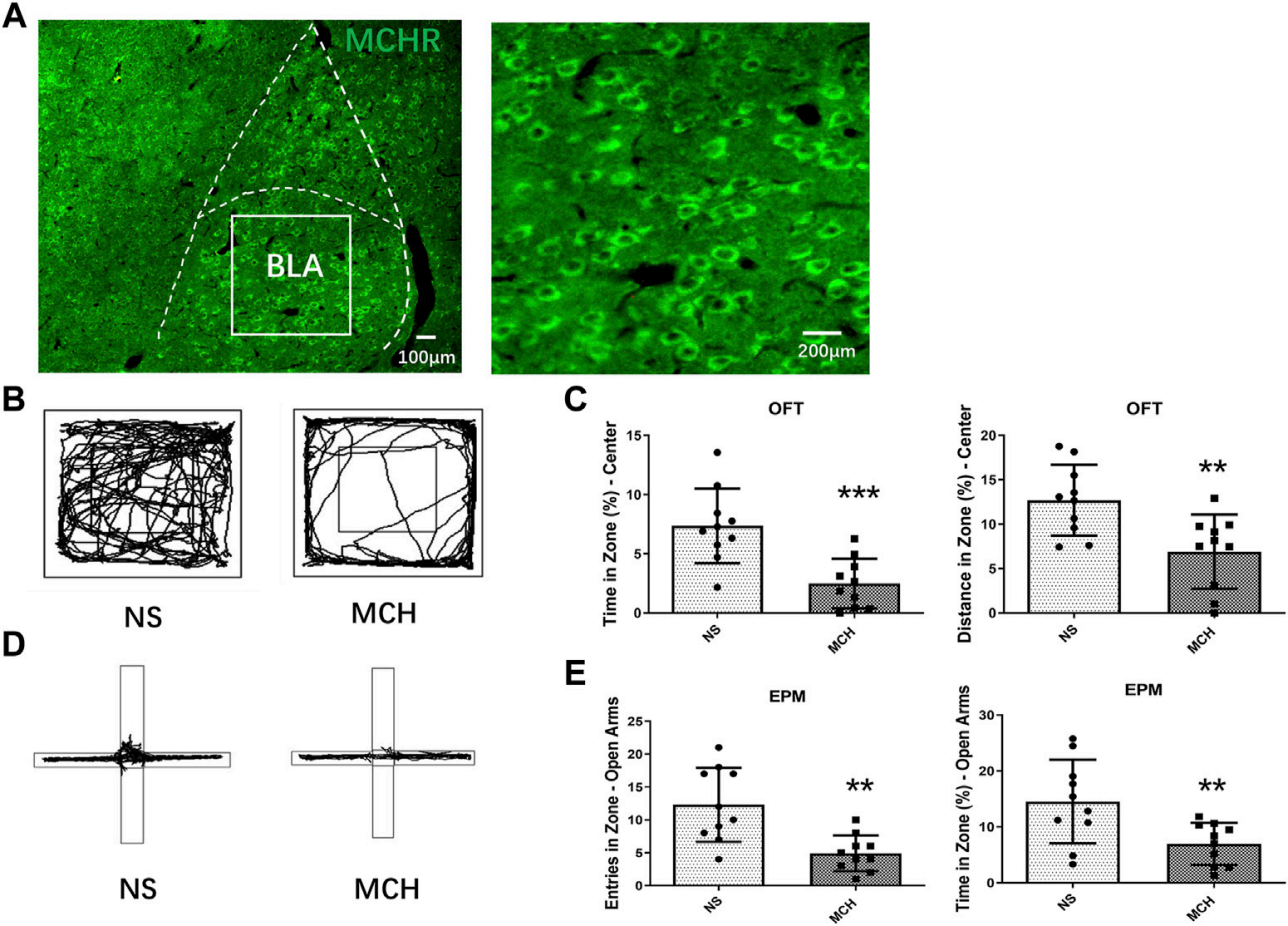
MCH peptide microinjection into the BLA induced anxiety-like behaviors in mice. **(A)** Fluorescent immunohistochemistry showed MCHR in the BLA. **(B)** Representative traces of locomotor activity in the OFT. **(C)** Effects of injecting the MCH peptide into the BLA on the center residence time and distance in the.OFT. **(D)** Representative traces of locomotor activity in the EPM. **(E)** Effects of injecting the MCH peptide into the BLA on the open arm entry times and residence time in the EPM. The data are presented as the mean ± SEM (*n* = 10 mice per group). *p < 0.05, **p < 0.01, ***p < 0.001 versus the NS group.

Fluorescence immunohistochemical results showed that MCHR was distributed in the BLA (Figure 1A). The experimental group was injected with MCH peptide, and the control group was injected with the NS. Behavioral tests were performed 30 min after the drug was injected. The time (*p* < 0.001, *n* = 10) and movement distance (*p* < 0.01, *n* = 10) in the OFT central zone were significantly shorter in the MCH group than in the control group (Figures 1B,C). In the EPM test, the mice in the MCH group entered the open arms significantly less often (*p* < 0.01, *n* = 10) and spent significantly less time there (*p* < 0.01, *n* = 10) than those in the control group (Figures 1D,E).

### Melanin-concentrating hormone-immunoreactive neurons in the lateral hypothalamic area projected to the basolateral amygdala

**FIGURE 2:**
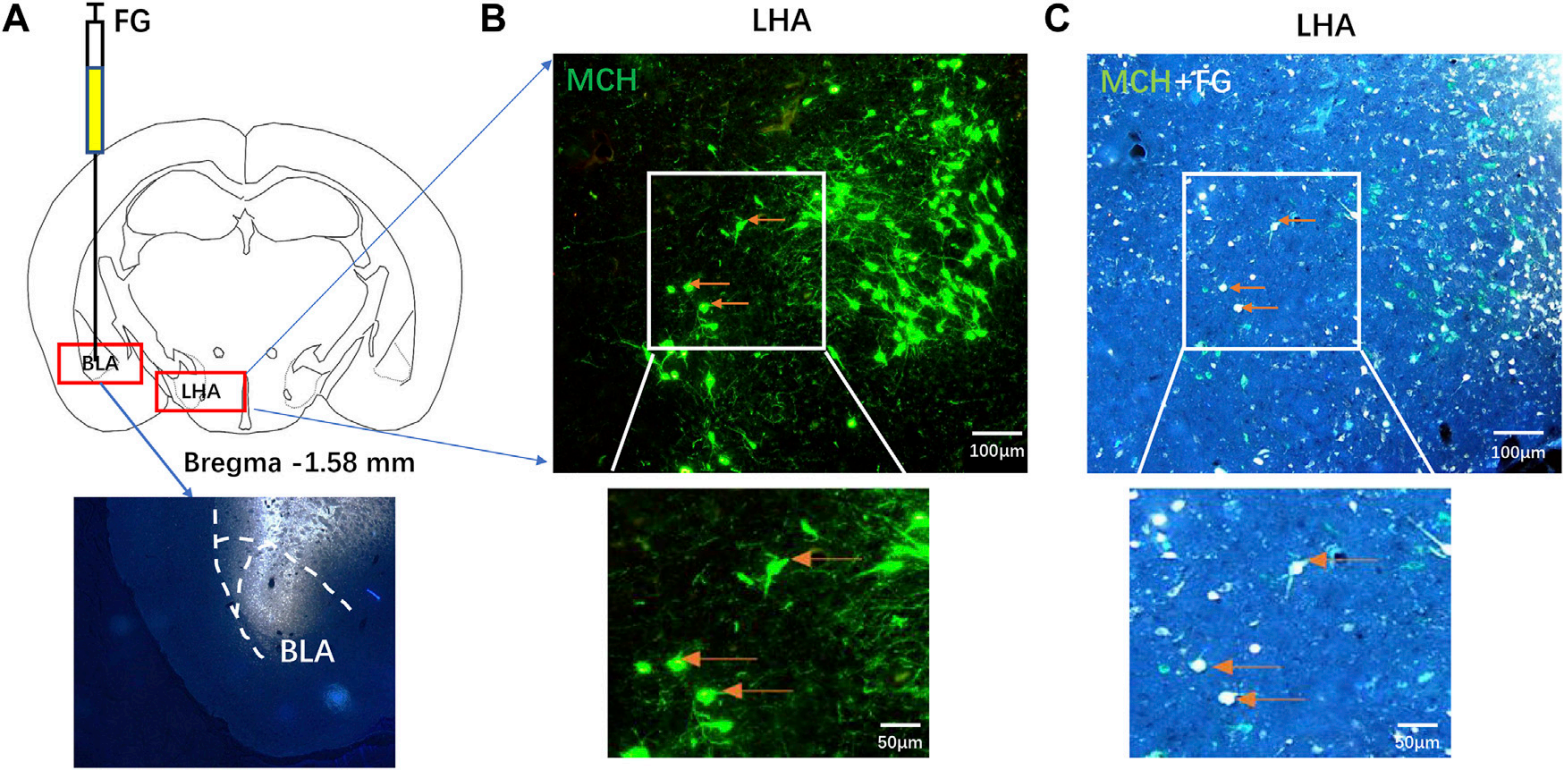
MCH-immunoreactive neurons in the LHA projected to the BLA. **(A)** A schematic diagram and injection site of FG microinjection into the BLA. **(B,C)** After seven days of FG retrograde tracking, the fluorescence immunohistochemistry showed that the MCH neurons in the LHA **(B)** and the MCH/FG co-expressing neurons extend from the BLA into the LHA **(C)** (*n* = 6).

FG was microinjected into the BLA of mice (Figure 2A). Microscopically, MCH-immunoreactive neurons were found in the LHA (Figure 2B), 32.15% ± 6.83% of MCH-labeled neurons contain FG, and 21.33% ± 5.32% of FG-labeled neurons showed immunoreactive MCH expression (Figure 2C), suggesting that some MCH-immunoreactive neurons in the LHA projected nerve fibers to the BLA.

### Chemogenetic activation of melanin-concentrating hormone neurons induced anxiety behaviors in mice

**FIGURE 3:**
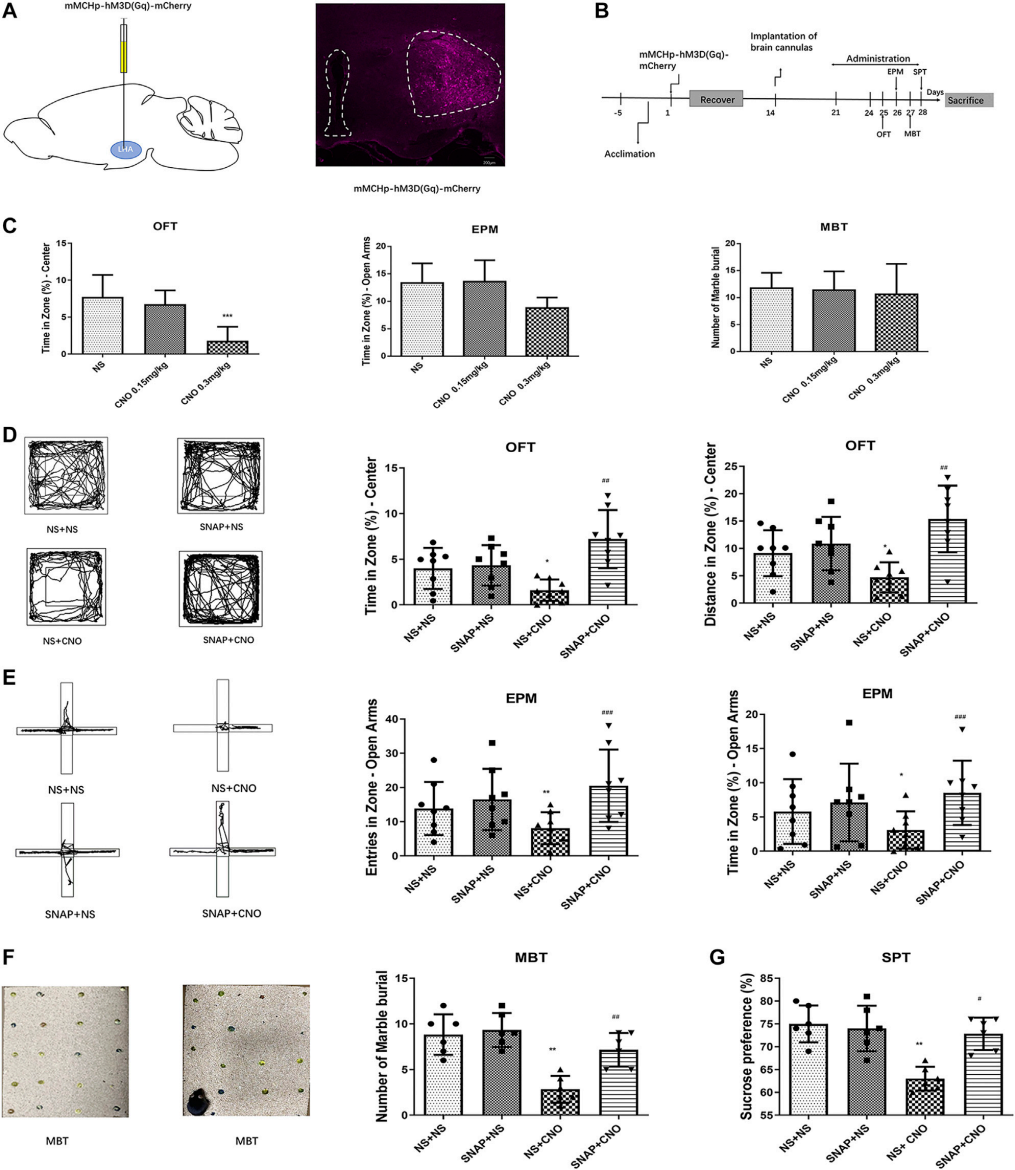
Chemogenetic activation of MCH neurons induced anxiety-like behaviors in mice. **(A)** A schematic diagram of an mMCHp-hM3D(Gq)-mCherry injection into the LHA (left) and a representative photograph of mMCHp-hM3D(Gq)-mCherry fluorescence at the injection site in the LHA (right). **(B)** An experimental procedure to activate the virus during behavioral experiments. After the mice had acclimated to the environment, the virus was injected into the LHA, with a cannula buried in the BLA; one week after their recovery from the surgery, the virus was activated by an intraperitoneal injection of CNO, and the MCHR1 blocker SNAP was injected into the BLA to measure the behavior of the mice. **(C)** Effects of 0.3 mg/kg and 0.15 mg/kg CNO on OFT, EPM and MBT in wild type mice. (*n* = 6–8 mice per group). **(D,E,F,G)** Representative traces of locomotor activity in the OFT **(D)**, EPM **(E)**, and MBT **(F)** (left), the effects of BLA injection of the MCHR1 blocker SNAP and intraperitoneal injection of CNO to activate the virus on the OFT center residence time and distance **(D)**, EPM open arm entry times and residence time **(E)**, the number of marbles buried in the MBT **(F)**, and sucrose preference **(G)** (right). The data are presented as the mean ± SEM (*n* = 6–8 mice per group). *p < 0.05, **p < 0.01, ***p < 0.001 versus the NS + NS group, #*p* < 0.05, ##*p* < 0.01, ###*p* < 0.001 versus the NS + CNO group.

The LHA of mice was injected with mMCHp-hM3D(Gq)-mCherry (Figure 3A). A cannula was then implanted into the BLA, After the recovery, the mice were tested OFT on day 25, EPM on day 26, MBT on day 27, and SPT on day 28 (Figure 3B). To rule out an effect of CNO on behavior, dose comparison was performed. The results showed that CNO in a dose of 0.15 mg/kg had no effect on the behavior of normal mice compared with the NS control group. A higher dose of 0.3 mg/kg CNO had no significant effect on the behavior of embedding beads in MBT, and had a tendency to reduce the duration of open arm stay in EPM, while this dose in OFT could significantly reduce the duration of stay in the center of mice (*p* < 0.001, *n* = 6–8; Figure 3C). Therefore, 0.15 mg/kg was selected for the activation of MCH neurons in the LHA.

The mice with AAV2/9-mMCHp-hM3D(Gq)-mCherry-WPRE-PA were divided into four groups: NS + NS, SNAP + NS, NS + CNO, and SNAP + CNO. Compared with the NS + NS group, the NS + CNO group had a shorter average time (*p* < 0.05, *n* = 8) and distance covered (*p* < 0.05, *n* = 8) in the OFT central zone (Figure 3D), fewer entries into the open arms (*p* < 0.01, *n* = 8) and less time spent there (*p* < 0.05, *n* = 8) in the EPM (Figure 3E), fewer buried beads (*p* < 0.01, *n* = 6) in the MBT (Figure 3F), and less preference for sucrose (*p* < 0.01, *n* = 6) in the SPT (Figure 3G).

A comparison between the SNAP + CNO and NS + CNO groups showed that the former had significantly longer times (*p* < 0.01, *n* = 8) and distances covered (*p* < 0.01, *n* = 8) in the OFT center (Figure 3D), more frequent entries (*p* < 0.001, *n* = 8) and longer times (*p* < 0.001, *n* = 8) in the open arms of the EPM (Figure 3E), more buried beads (*p* < 0.01, *n* = 6) in the MBT (Figure 3F), and a greater preference for sucrose (*p* < 0.05, *n* = 6) in the SPT (Figure 3G). These results showed that the activation of MCH neurons caused anxiety-like responses in mice, whereas SNAP microinjection into the BLA alleviated them.

### Chronic acute combining stress induced anxiety behaviors and activation of melanin-concentrating hormone neurons in the mice

**FIGURE 4:**
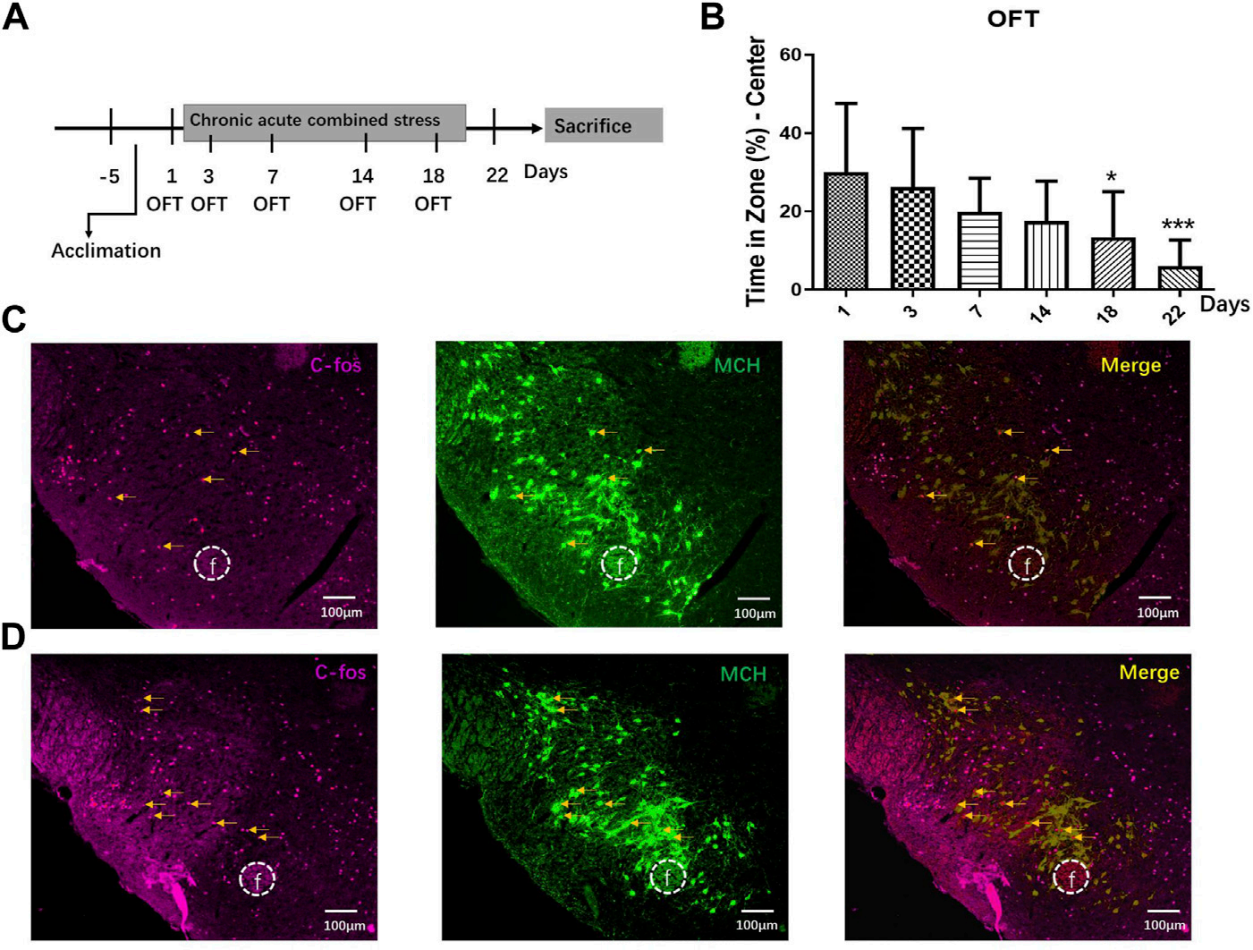
CACS induced anxiety-like behaviors and activated MCH neurons in the mice. **(A)** The timeline of Experiment 4. **(B)** Changes in stay time at the OFT center on days 1, 3, 7, 14, 18, and 22 during CACS modeling and CACS mice showed anxiety-like behaviors (*n* = 10). **(C)** A representative photograph of MCH/C-FOS fluorescence immunohistochemistry in the LHA of the control group and neurons co-expressed with MCH/C-FOS, indicating the number of MCH neurons activated (*n* = 6). **(D)** Representative fluorescence immunohistochemistry photographs of MCH/C-FOS in the LHA of the CACS model mice (*n* = 6).

Anxious mouse models were created by combining acute and chronic stress for 22 days (Figure 4A). The CACS mice spent significantly less time in the OFT central zone than normal mice on Day 18 and later (*p* < 0.05, *n* = 10; Figure 4B). Acute stress was induced for 2 h before sacrifice on Day 22, and six mice were then perfused and fixed to obtain frozen sections. The brains of the normal and anxious mice were sliced for MCH and c-Fos fluorescent immunohistochemical staining. Microscopically, MCH- and c-Fos-immunoreactive neurons were observed in the LHA, and some MCH neurons showed c-Fos-positive expression (Figures 4C,D). A comparison of c-Fos immunoreactive expression in the LHA of normal and anxious mice showed significantly more numerous c-Fos and MCH double-labeled cells in the MCH positive neurons of anxious mice than in that of normal mice (27.53% ± 5.32% vs. 7.67% ± 1.81%; *p* < 0.05, *n* = 6), suggesting increased activation of MCH neurons in anxious mice.

### SNAP microinjection into the basolateral amygdala alleviated anxiety-like behaviors in chronic acute combining stress mice

**FIGURE 5:**
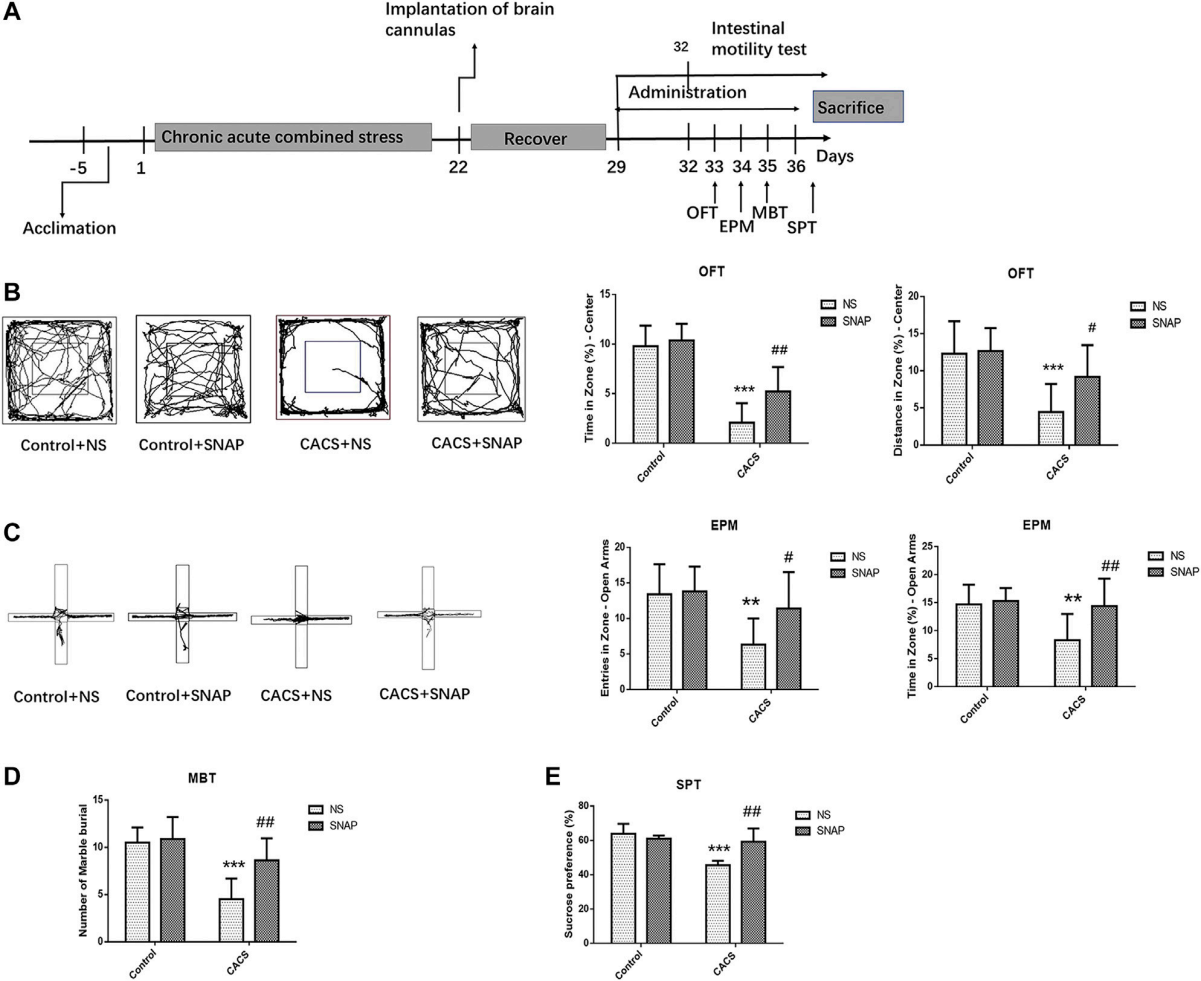
SNAP microinjection into the BLA affected the anxiety-like behaviors of CACS mice. **(A)** The timeline of Experiment 5. **(B,C,D,E)** Representative traces of locomotor activity in the OFT **(B)** (left), EPM **(C)** (left), the effects of microinjection of SNAP into the BLA of the CACS model mice on the OFT center residence time and distance **(B)**, EPM open arm entry times and residence time **(C)**, the number of marbles buried in the MBT **(D)**, and sucrose preference **(E)**. The data are presented as the mean ± SEM (*n* = 6–10 mice per group). *p < 0.05, **p < 0.01, ***p < 0.001 versus the Control + NS group, #*p* < 0.05, ##*p* < 0.01, ###*p* < 0.001 versus the CACS + NS group.

A cannula was then implanted into the BLA of the CACS mice on Day 22, and behavioral tests and intestinal motility test were administered after a seven-day recovery (Figure 5A). Both CACS mice and normal control mice were injected with SNAP or NS in BLA, and behavioral experiment was tested 30 min after injection. Compared with the Control + NS group, the CACS + NS had a shorter time (*p* < 0.001, *n* = 10) and distance (*p* < 0.001, *n* = 10) in the OFT central zone (Figure 5B), less frequent entries (*p* < 0.01, *n* = 10) and shorter times (*p* < 0.01, *n* = 10) in the open arms of the EPM (Figure 5C), less buried beads (*p* < 0.001, *n* = 8) in the MBT (Figure 5D), and a less preference for sucrose (*p* < 0.001, *n* = 6) in the SPT (Figure 5E). After SNAP was injected into BLA of Control mice, no significant changes were observed in behavioral tests compared with Control + NS group (*p* > 0.05, *n* = 6–10).

The behavior of CACS mice was measured 30 min after microinjection of SNAP, the time (*p* < 0.01, *n* = 10) and distance covered (*p* < 0.05, *n* = 10) in the OFT central zone were significantly greater in CACS + SNAP mice than in CACS + NS mice. (Figure 5B). The CACS + SNAP mice also showed significantly more entries (*p* < 0.05, *n* = 10) and longer times (*p* < 0.01, *n* = 10) in the open arms of the EPM than CACS + NS mice (Figure 5C). Furthermore, the CACS + SNAP group had more buried marbles (*p* < 0.01, *n* = 8; Figure 5D) and a greater sucrose preference (*p* < 0.01, *n* = 6; Figure 5E) than the CACS + NS group.

### SNAP microinjection into the basolateral amygdala affected the intestinal characteristics of chronic acute combining stress mice

**FIGURE 6:**
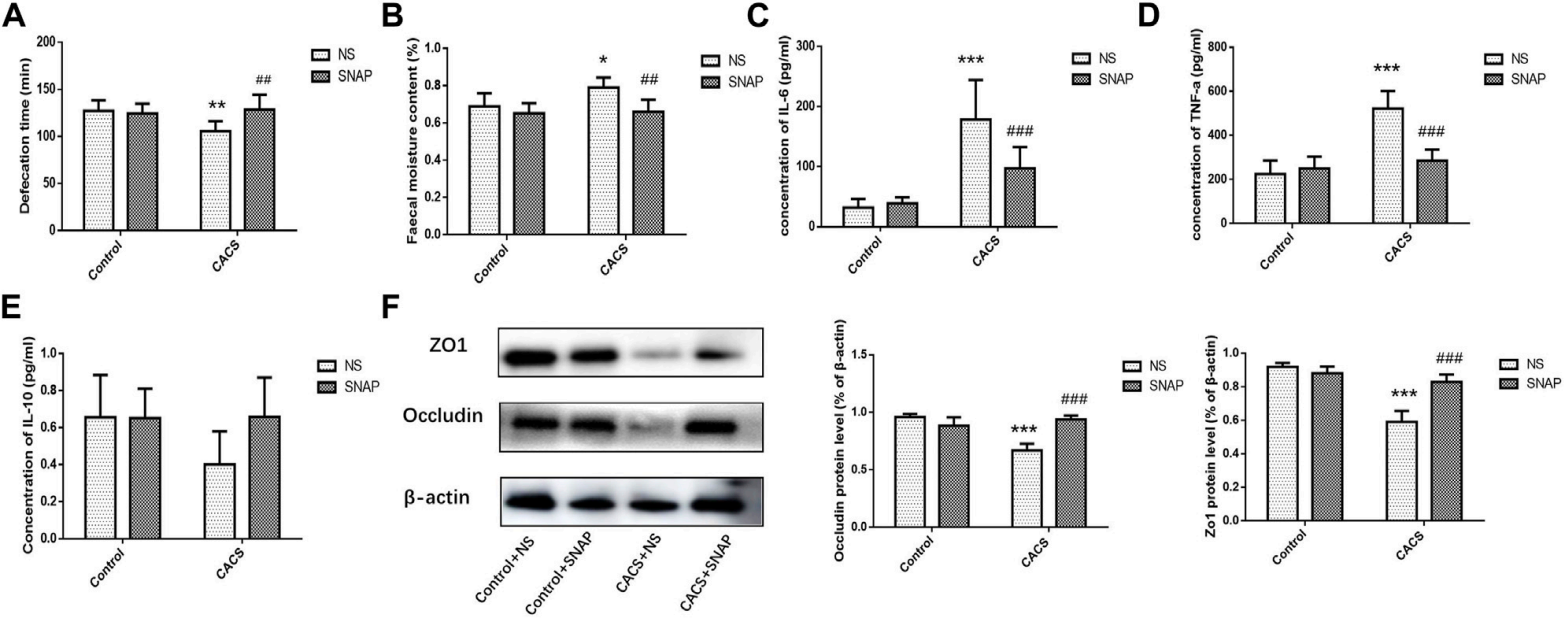
Microinjection of SNAP in BLA affected intestinal characters in CACS mice. **(A,B)** Changes in fecal excretion time **(A)**, fecal water content **(B)**, and the effects of injecting SNAP into the BLA on fecal excretion time and fecal water content. **(C,D,E)** Changes in IL-6, TNF-α, and IL-10 in the colon tissues of the CACS model mice and the effects of injecting SNAP in the BLA on IL-6, TNF-α, and IL-10 in the colon tissues of the CACS model mice. **(F)** Changes in occludin and ZO1 in the colons of the CACS model mice and the effect of injecting SNAP in the BLA on occludin and ZO1 in the colons of the CACS model mice. The data are presented as the mean ± SEM (*n* = 6–10 mice per group). *p < 0.05, **p < 0.01, ***p < 0.001 versus the control group, #*p* < 0.05, ##*p* < 0.01, ###*p* < 0.001 versus the CACS + NS group.

The Control + NS group, Control + SNAP group, CACS + NS group and CACS + SNAP group were injected with NS or SNAP in BLA and then gavaged with charcoal solution to measure fecal excretion time and water content. Compared with the Control + NS group, the fecal excretion time was significantly shorter (*p* < 0.01, *n* = 8, Figure 6A), and the fecal water content was significantly higher (*p* < 0.05, *n* = 8, Figure 6B) in the CACS + NS mice. However, the CACS mice receiving SNAP microinjection into the BLA exhibited significantly longer fecal excretion times (*p* < 0.01, *n* = 8, Figure 6A) and lower fecal water contents (*p* < 0.01, *n* = 8, Figure 6B) than the CACS + NS mice. This suggests that SNAP alleviated diarrhea symptoms in the CACS model mice. ELISA showed that TNF-α and IL-6 expression was significantly higher in the collected colon tissues of CACS + NS mice (*p* < 0.001, *n* = 10, Figures 6C,D) than in the Control + NS mice, while IL-10 expression did not differ significantly (*p* > 0.05, *n* = 8, Figure 6E). In CACS mice treated with SNAP, expressions of TNF-α and IL-6 were decreased significantly in the colon compared with CACS + NS mice (*p* < 0.001, *n* = 10, Figures 6C,D). The western blot results showed that the expression of occludin and ZO1 was significantly lower in the colon tissues of CACS + NS mice than in the Control + NS mice and significantly higher in the CACS + SNAP mice than in the CACS + NS mice (*p* < 0.001, *n* = 6, Figure 6F).

### SNAP microinjection into the basolateral amygdala did not modify the expression of neuronal nitric oxide synthase or 5-HT in chronic acute combining stress mice

**FIGURE 7:**
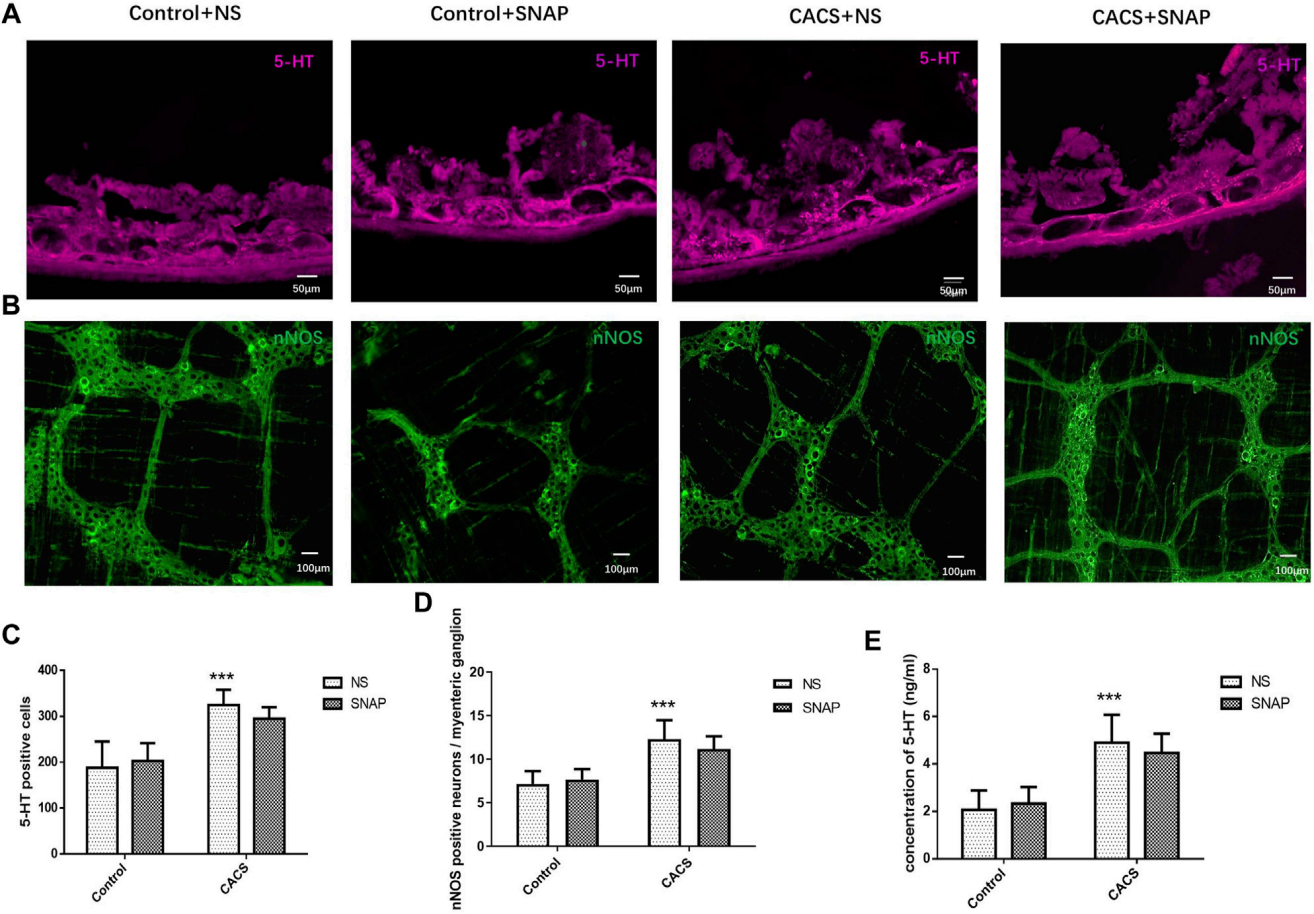
SNAP microinjection into the BLA did not modify the expression of nNOS or 5-HT in CACS mice. **(A,C,E)** Effects of SNAP microinjection into the BLA on 5-HT expression in the colons of the CACS model mice. The number of 5-HT-positive cells and concentration increased in the model group but did not decrease significantly in the SNAP group. **(B,D)** Effects of SNAP microinjection into the BLA on nNOS expression in the colons of the CACS model mice. The number of nNOS-positive cells increased in the model group but did not decrease significantly in the SNAP group. The data are presented as the mean ± SEM (*n* = 6–8 mice per group). *p < 0.05, **p < 0.01, ***p < 0.001 versus the control group, #*p* < 0.05, ##*p* < 0.01, ###*p* < 0.001 versus the CACS + NS group.

The myenteric plexus of the intestine was exposed for fluoro-immunohistochemical staining. The results showed significantly higher 5-HT expression in the CACS + NS group than in the Control + NS group (*p* < 0.001, *n* = 6–8, Figures 7A,C,E). The expression of 5-HT did not differ significantly between the CACS + NS and CACS + SNAP groups (*p* > 0.05, *n* = 6–8, Figures 7A,C,E). Likewise, the expression of nNOS was higher in the CACS + NS group than in the Control + NS group (*p* < 0.001, *n* = 6, Figures 7B,D) but did not differ significantly between the CACS + NS and CACS + SNAP groups (*p* > 0.05, *n* = 6, Figures 7B,D).

## Discussion

In this study, both exogenous MCH microinjected into the BLA and chemogenetic activation of MCH neurons in the LHA induced anxiety-like behaviors, and the MCHergic circuit from the LHA to the BLA was investigated in mice. The hyper- reactivity of MCHergic circuit might play an important role in inducing anxiety in the CACS mice, which was verified by microinjection of MCHR1 blocker SNAP into the BLA. Further, intestinal disturbances associated with anxiety were alleviated by administration of SNAP in the BLA through reducing inflammation and improving intestinal permeability.

Considerable evidence suggests that the LHA is a hub for the integration and regulation of feeding, reward, stress, and motivated behavior (Hurley and Johnson, 2014; Tyree et al., 2018; Perez-Bonilla et al., 2020). The gut-brain peptide MCH, mainly expressed in the LHA is associated with some of these functions, including energy balance and sleep–wake rhythms, anxiety-like behaviors and so on (Pelluru et al., 2013; Oh et al., 2020; Al-Massadi et al., 2021). Kim et al. (2015) found that transcriptionally upregulated MCH expression induced by repeated stress promoted anxiety-like behaviors, and that the key nucleus involved in the response seems to be the BLA (Kim and Han, 2016c). As known, the BLA is the “fear and stress center” of the limbic system and expresses MCHR1 (Jang J. H. et al., 2018), which suggests a possible functional link between the LHA and the BLA areas in anxiety and related symptoms.

Firstly, our fluorescence immunohistochemistry results showed the expression of MCHR in the BLA, and then we microinjected exogenous MCH into the BLA of mice and examined their behaviors using the OFT and EPM test 30 min later. The observed anxiety-like behaviors led us to speculate that there might be a fiber projection relationship between MCHergic neurons from the LHA and BLA. To determine the origin of the nerve fibers and prove this conjecture, we then injected FG into the BLA of mice for retrograde tracking. The fluorescence immunohistochemistry results showed that FG-labeled neurons in the LHA partially coincided with MCH neurons, indicating that MCHergic neurons in the LHA could project to the BLA. It has been reported that stress-induced activation of MCH receptors in the BLA can lead to deficits in social and emotion-related behaviors (Kim and Han, 2016b). Furthermore, injecting MCH into the BLA has been found to generally increase p-CamKIIα and produce socially impaired and depressive behaviors. Conversely, MCH knockout has been shown to reduce p-CAMKIIα in the BLA of mice and to make them resistant to chronic stress (Kim et al., 2015). Our results are consistent with mentioned findings.

We further activated MCH neurons in the LHA using chemogenetics to investigate the role of endogenous MCH in anxiety-like behavior. We induced MCH neuron activation by intraperitoneal injection of CNO, a ligand of hM3Dq, in AAV-injected mice. Because CNO is one of the metabolites of clozapine, which belongs to a class of antipsychotic drugs, we first determined the appropriate concentration in mice to rule out an effect of the drug itself on anxiety. Referring to previous studies (Naganuma et al., 2018), we first chose a dose of 0.3 mg/kg. However, our experiment showed that this dose affected the behavior of mice in OFT, whereas a dose of 0.15 mg/kg had no significant effect. Thus, we activated MCH neurons in the LHA with 0.15 mg/kg, which aggravated anxiety. Conversely, blocking the MCH receptors in the BLA ameliorated it. These results indicate that MCH neurons play a role in anxiety-like behaviors through the LHA–BLA pathway.

It was reported that MCH receptors are expressed in glutamate and GABAergic neurons of BLA (Kim et al., 2015). So, on the one hand, glutamate neurons in the BLA were speculated to be directly regulated by MCH from LHA. Since BLA sends out glutamate energy projection to CeA, CeA in turn projects adrenocorticotropin-releasing hormone to the paraventricular nucleus of hypothalamus to participate in the activation of HPA axis, thus regulating stress-induced anxiety behavior and intestinal changes (Flandreau et al., 2012). On the other hand, based on the networks of parvalbumin-positive interneurons in the BLA (Woodruff and Sah, 2007) and increased locomotor activity led by MCH receptor 1 deletion from GABAergic neurons (Chee et al., 2019), it is believed that GABAergic interneurons indirectly alter the excitability of glutamatergic projection neurons.

Although chronic mild stress model has been reported to produce diverse modifications, it has been widely used to study behaviours associated with anxiety and depression and possesses face, construct, and predictive validity (Willner, 1997; Wiborg, 2013; Vitale et al., 2017). Chronic unpredictable and restraint stress can mimic anxiety states and irritable bowel syndrome (IBS)-like symptoms to a considerable extent, ranging from central nervous system disorders to bowel movement and visceral sensory disturbance (Zou et al., 2008). In this study, mice subjected to 22 days of CACS showed anxiety-like behaviors in the OFT and EPM test. In frozen sections of their brains, c-Fos expression was significantly higher in CACS model mice than in control mice, indicating greater MCH neurons activation. Conversely, microinjection of an MCH receptor antagonist into the BLA relieved the anxiety-like behaviors in the OFT and EPM test in CACS model mice and led to increases in buried marbles and sucrose preference.

Stress-induced IBS-like symptoms mainly include changes in stool frequency or form due to visceral sensitivity and abnormal intestinal motility (Jia et al., 2018). IBS is thought to be a disorder of the brain–gut axis, which is the pathway through which the intestine communicates with the brain through neuroendocrine and inflammatory pathways (Videlock and Chang, 2021). Kokkotou et al. (2008) detected increased expression of MCH and its receptor in the colon of patients with inflammatory bowel disease, but its role in the intestinal tract remains unclear. In this study, we detected changes in the fecal excretion time and fecal water content in CACS mice on Day 22, suggesting that anxiety caused significant intestinal dysfunction in mice. Conversely, SNAP microinjected into the BLA ameliorated IBS-like symptoms, further confirming the role of the LHA–BLA MCHergic pathway in stress-induced IBS-like symptoms.

Clinical studies have shown increased number and activation of mast cells in patients with IBS, which release various mediators, including pro-inflammatory cytokines and 5-HT, to alter intestinal sensation, motility, secretion, and permeability (Krammer et al., 2019). In this study, we also found higher expression levels of 5-HT in CACS group showed by intestinal immunohistochemistry and ELISA. It is believed that the abnormal expression of 5-HT might be a factor to influence the intestine of CACS mice. NO synthase (NOS) is also a factor involved in the pathogenesis of IBS. Previous studies showed that the expression of nNOS protein and mRNA in the colon of neonatal maternal separation model rats was higher than that of normal rats, while inducible NOS (iNOS) and endothelial NOS (eNOS) did not change significantly in the two groups (Tjong et al., 2011). In addition, nNOS is a biomarker of Cajal stromal cells in IBS-like diarrhea caused by stress (Jang D. E. et al., 2018). The increased nNOS suggested the presence of more Cajal cells in CACS mice. Other studies have also shown that nNOS is positively correlated with irritable bowel motility (Han et al., 2021). However, SNAP treatment had no significant effect on the expression of 5-HT or nNOS in CACS mice, which implied that neither 5-HT nor nNOS might be the mediator through which the LHA–BLA MCHergic neural pathway acts on the gastrointestinal tract.

It is known that low-grade inflammation and changes in intestinal permeability are frequent among IBS patients (Jang J. H. et al., 2018). We further investigated the intestinal permeability and inflammation factors in CACS mice. Western blot analysis of tight junction–related proteins showed that the expression of occludin and ZO1 decreased significantly in CACS mice but increased after SNAP treatment. Furthermore, ELISA detection of TNF-α and IL-6 showed elevated levels of both inflammatory factors in CACS mice. The intestinal barrier prevents antigens, pathogens, and other pro-inflammatory substances from entering the body. However, increased intestinal permeability can lead to local or systemic inflammation and disease (Fukui, 2016). SNAP reduced the expression of inflammatory factors in the model group, inhibiting intestinal inflammation. Therefore, we speculate that BLA-blocking MCHR1 could reduce intestinal hyperpermeability and inflammation caused by stress, thus improving intestinal motility.

SNAP, as the specific antagonist of the MCHR1, is effective in reducing MCH-induced food-seeking behaviors and high-fat food reinforced operant responding (Nair et al., 2009). In CACS mice, the pretreatment of SNAP in the BLA ameliorated IBS-like symptoms, increased the expression of occludin and ZO1 and reduced the expression of inflammatory factors, which suggested that MCHR1 was involved in stress-inducing intestinal symptoms. Similar results also reported SNAP attenuated stress-induced hyperthermia and reversed decreased sucrose intake in the chronic mild stress anhedonia model (Smith et al., 2009). However, SNAP did not recover the elevated expression of 5-HT or nNOS in CACS mice, which indicated different neurotransmitters might be responding to CACS inducing stress. It has been reported that oxytoxin, galanin and dopamine are involved in stress-related behavioral modulation (Bajo et al., 2012; de la Mora et al., 2016; Hernandez-Perez et al., 2018; Narvaez et al., 2018; Micioni Di Bonaventura et al., 2019; Ballas et al., 2021; Deal et al., 2021).

**FIGURE 8:**
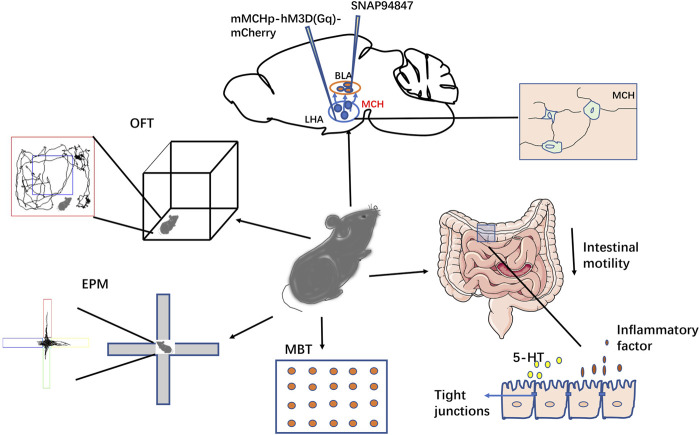
Schematic of this study. This study investigated the role of the LHA-BLA MCHergic neural pathway in regulating stress-induced anxiety and intestinal function in mice. The results showed that both injection of MCH and activation of MCH neurons in the BLA could lead to anxious-like behavior in mice, while injection of SNAP into the BLA could reduce anxious-like behavior in both activated MCH neurons and CACS model mice. In addition to the changes in their emotional behavior, the CACS model mice showed intestinal IBS-like symptoms (inflammation and increased permeability). SNAP treatment could improve these symptoms, suggesting that this pathway is involved in regulating emotional behavior and intestinal motility changes in anxious mice.

In conclusion, our findings suggest that MCH plays a role in stress-induced anxious behaviors and intestinal dynamic changes through the LHA–BLA pathway. The MCHR1 antagonist SNAP acting on the BLA can relieve stress-induced anxiety and reduce intestinal permeability and inflammation, thus alleviating stress-induced IBS-like symptoms (Figure 8). However, the detailed molecular mechanisms remain unclear. Further research is needed to elucidate the role of MCH in anxiety disorders through the LHA–BLA pathway and explore potential IBS treatments based on the brain–gut axis.

## Data Availability

The original contributions presented in the study are included in the article/Supplementary Material, further inquiries can be directed to the corresponding author.

## Abbreviations

BLA, basolateral amygdala; CACS, chronic acute combining stress; CeA, central amygdala; CNO, Clozapine N-oxide; ELISA, enzyme-linked immune-sorbent assay; eNOS, endothelial nitric oxide synthase; EPM, elevated plus maze; 5- HT, 5-hydroxytryptamine; FG, Fluoro-Gold; IBS, irritable bowel syndrome; IL-6, interleukin-6; IL-10, interleukin-10; iNOS, inducible nitric oxide synthase; LHA, lateral hypothalamic area; MBT, marble burying test; MCH, melanin-concentrating hormone; MCHR, melanin-concentrating hormone receptor; nNOS neuronal nitric oxide synthase; NS, normal saline; OFT, open field test; PBS, phosphate buffer saline; PFA, paraformaldehyde; SNAP, SNAP-94847; SPT, sucrose preference test; TNF-α, tumor necrosis factor alpha.
